# Preparation and Preliminary Biological Evaluation of Novel ^99m^Tc-Labelled Thymidine Analogs as Tumor Imaging Agents

**DOI:** 10.3390/molecules17078518

**Published:** 2012-07-16

**Authors:** Chunxiong Lu, Quanfu Jiang, Cheng Tan, Jie Tang, Jiankang Zhang

**Affiliations:** 1 Key Laboratory of Nuclear Medicine, Ministry of Health-Jiangsu Key Laboratory of Molecular Nuclear Medicine, Jiangsu Institute of Nuclear Medicine, Wuxi 214063, China; 2 School of Chemical and Material Engineering, Jiangnan University, Wuxi 214122, China

**Keywords:** thymidine derivative, ^99m^Tc-labelled, biodistribution, tumor imaging

## Abstract

Two kinds of novel thymidine derivatives, *N*-thymidine-yl-*N′*-methyl-*N′*-{*N′′*-[2-sulfanyl-(ethylamino)acetyl]-2-aminoethylsulfanyl-1-hexanamide}-ethanediamine (TMHEA) and *N*-thymidine-yl-*N′*-methyl-*N′*-{*N′′*-[2-sulfanyl-(ethylamino)acetyl]-2-aminoethylsulfanyl-1-hexanamide}-hexanediamine (TMHHA) were prepared and successfully labeled with ^99m^Tc in high labeling yields. The *in vitro* stability and *in vivo* biodistribution of ^99m^Tc-TMHEA and ^99m^Tc-TMHHA were investigated and compared. The biodistribution studies indicate that the radiotracer ^99m^Tc-TMHEA displays selective tumor uptake, suggesting it is a potential tumor imaging agent.

## 1. Introduction

In clinical oncology, 2′-deoxy-2′-[^18^F]fluoro-D-glucose (^18^F-FDG), a glucose derivative, has been widely used in recent years for tumor imaging with positron emission tomography (PET). However, ^18^F-FDG is a non-specific tracer for tumor imaging since glucose is highly utilized by many other cells, such as macrophages found in inflammatory lesions [1,2]. To overcome this inconvenience of ^18^F-FDG, many studies have focused on the development of a variety of DNA precursors [1,3,4,5]. Specifically, labeled thymidine analogs can target the proliferative activity of malignant lesions [6,7], and several useful ligands, such as ^11^C-labeled nucleoside thymidine [1], 3′-deoxy-3′-[^18^F]fluoro thymidine (^18^F-FLT) [1,3,4,5] and its analog ^18^F-FMAU [8] have demonstrated their good imaging features. However, these tracers were labeled with either ^11^C or ^18^F, which are short half-life isotopes produced by a cyclotron, with complicated radiochemical synthesis and the lower radiochemical yield and high cost of PET examination, all of which limit their use as tracers in routine clinical studies.

Technetium-99m (^99m^Tc), the most commonly used radioisotope in SPECT, is continuously available at a reasonable cost in many hospitals and has ideal nuclear properties for imaging (T_1/2_ = 6.02 h, γ = 140 keV). Therefore it is important to develop a ^99m^Tc labeled thymidine analog so as to provide the ideal characteristics needed for routine clinical studies [9,10,11]. In the previous work of our group, a series of technetium-99m labeled thymine derivatives have been prepared and their *in vivo* biological properties were systematically investigated [12,13]. It was found that the uptake ratio of tumor to muscle of ^99m^Tc-NHT was higher than that of ^99m^Tc-ANMdU, which means that uptake ratio of tumor to muscle maybe increase with increasing carbon chain length between the thymidine and N_2_S_2_ ligand. However, to the best of our knowledge, extension and optimization of the linker chain between the thymidine and N_2_S_2_ ligand to develop novel tumor imaging agent has been largely unexplored.

**Figure 1:**
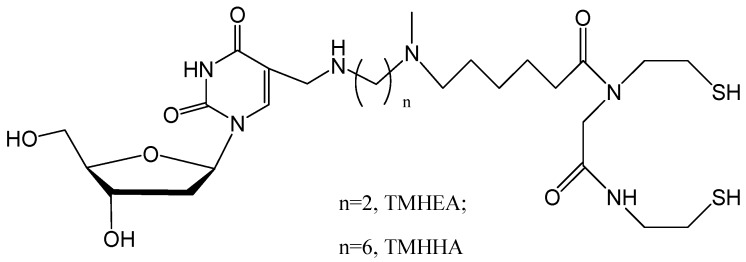
Structures of TMHEA and TMHHA.

For the purpose of developing novel tumor imaging agents with excellent biological properties, we have continued to extend the number of methylene units between the thymidine and N_2_S_2_ ligand. In this work, two novel ^99m^Tc-labeled thymidine derivatives were prepared and reported, *i.e*., ^99m^Tc-TMHEA and ^99m^Tc-TMHHA (Figure 1). Their *in vitro* stability and *in vivo* biodistribution were also studied.

## 2. Results and Discussion

### 2.1. Chemistry and Radiolabeling

TMHEA and TMHHA were synthesized by seven step reactions from the starting materials ethanediamine and hexanediamine, respectively. The target compounds were identified by MS, ^1^H-NMR and ^13^C-NMR, and the results agreed well with the expected chemical structures. ^99m^Tc-TMHEA and ^99m^Tc-TMHHA were labeled with Na^99m^TcO_4_ by reduction with stannous chloride. For TLC analysis, with the toluene/acetonitrile/methanol (v/v/v = 3/1/1) system, the R*_f_* values of ^99m^Tc-TMHEA and ^99m^Tc-TMHHA were about 0.7–0.8, while ^99m^Tc-colloidal impurities remain at 0–0.1.

**Figure 2:**
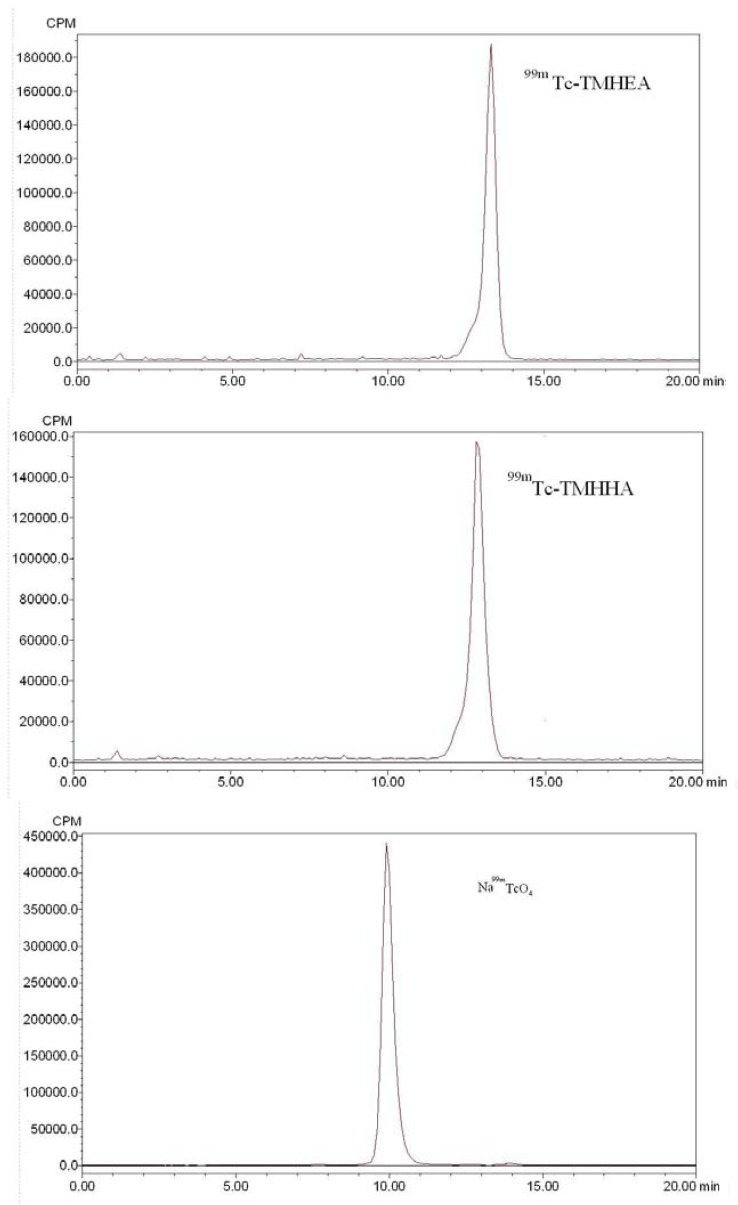
HPLC chromatograms (^99m^Tc-TMHEA t_R_ = 13.3min, ^99m^Tc-TMHHA t_R_ = 12.8min and ^99m^TcO_4_^−^ t_R_ = 9.9min).

HPLC analysis revealed the formation of free technetium (Na^99m^TcO_4_) that was eluted at a retention time of 9.9 min, whereas ^99m^Tc-TMHEA and ^99m^Tc-TMHHA eluted at retention times of 13.3 min and 12.8 min, respectively (Figure 2). For each radiolabeled complex, the single peak in the HPLC-chromatogram clearly shows the formation of only one complex and excludes the possibility of residual Na^99m^TcO_4_ or other components. That is, the chelation of technetium with the N_2_S_2_ is unique and complete.

According to the TLC and HPLC analysis, the radiochemical purities of ^99m^Tc-TMHEA and ^99m^Tc-TMHHA were all greater than 95%. The radiolabeled compounds were used immediately after the formulation for both *in vitro* and *in vivo* studies.

### 2.2. *In vitro* Stability and Octanol-Water Partition Coefficient

The *in vitro* stabilities of ^99m^Tc-TMHEA and ^99m^Tc-TMHHA were performed in PBS (pH = 7.4) for different time intervals (1, 2, 3, 4, 5, 6 h) at 37 °C. The stability was presented as RCP on the basis of the HPLC analysis. After 6 h of incubation, more than 95% of ^99m^Tc-TMHEA and ^99m^Tc-TMHHA remained intact in the PBS. The results indicate that the labeling efficiency of these complexes was high and their stability duration was long enough to allow further biodistribution and imaging studies.

The octanol-water partition coefficients (log*P*) for ^99m^Tc-TMHEA and ^99m^Tc-TMHHA were 1.01, 0.99 and 1.06, 1.02 in PBS at two different pH values of 7.0 and 7.4, respectively (see Table 1), which demonstrated that the longer the carbon chain, the smaller the logP, and the liposolubility at pH = 7.4 was higher than that at pH = 7.0. As well known, the log*P* value is a very useful parameter that can be used to understand the behavior of a drug and predict its distribution in the organism in combination with other parameters [14].

**Table 1:** Octanol-Water partition coefficient.

Constituent	pH = 7.0	pH = 7.4
^99m^Tc-TMHEA	1.01 ± 0.03	1.06 ± 0.02
^99m^Tc-TMHHA	0.99 ± 0.01	1.02 ± 0.03

### 2.3. Blood Kinetics Studies

**Figure 3:**
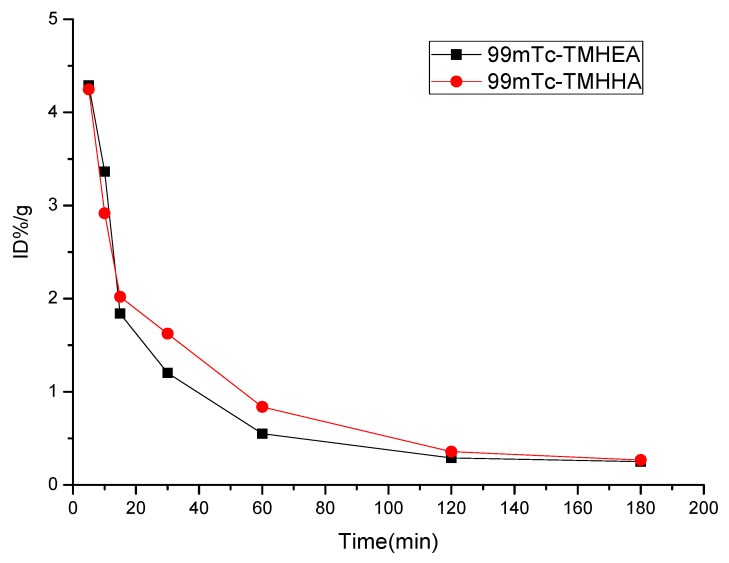
Pharmacokinetic curves in the mice for ^99m^Tc-TMHEA and ^99m^Tc-TMHHA.

Pharmacokinetic parameters are listed in Table 2. Figure 3 shows the blood clearance of ^99m^Tc-TMHEA and ^99m^Tc-TMHHA in the mice 3 h post injection. Pharmacokinetics of ^99m^Tc-TMHEA and ^99m^Tc-TMHHA comply with the two-compartment model with the pharmacokinetic equations of C = 5.24e^−0.11t^ + 1.14e^−0.02t^ and C = 5.51e^−0.21t^ + 2.44e^−0.02t^, respectively. The values of total body clearance (CL) were 0.10 and 0.09 and the area under the curve (AUC) were 162 and 184 for ^99m^Tc-TMHEA and ^99m^Tc-TMHHA, respectively. In the early phase, the blood clearance of ^99m^Tc-TMHHA was slower than ^99m^Tc-TMHEA. After 2 h, the radioactivity concentration of two tracer agents in blood reaches an equilibrium which coincides with the pharmacokinetic parameters CL, AUC and the pharmacokinetic curves.

**Table 2:** Pharmacokinetic parameters of the ^99m^Tc-TMHEA and ^99m^Tc-TMHHA in mice.

Parameters	^99m^Tc-TMHEA	^99m^Tc-TMHHA
K_12_ (min^−1^)	0.033	0.101
K_21_ (min^−1^)	0.035	0.082
K_e_ (min^−1^)	0.032	0.035
CL (%ID/g/min)	0.098	0.086
T_1/2α_ (min)	6.785	3.234
T_1/2β_ (min)	36.029	33.448
AUC (%ID/g/min)	162.658	186.894

### 2.4. Biodistribution Studies

Biodistributions of ^99m^Tc-TMHEA and ^99m^Tc-TMHHA were determined in tumor-bearing mice, and the data is shown in Table 3 as the percentage of administered activity (injected dose) per gram of tissue (%ID/g). Inspecting Table 3, one can observe that ^99m^Tc-TMHEA and ^99m^Tc-TMHHA are mainly accumulated in the kidney, bladder and liver, which means that the clearances of ^99m^Tc-TMHEA and ^99m^Tc-TMHHA are mainly through the renal pathway, and to a lesser extent, through the hepatobiliary pathway.

At 5 min post injection, the tumor uptake was 2.51 ± 0.28 and 2.38 ± 0.41 %ID/g, the muscle uptake was 1.93 ± 0.16 and 1.75 ± 0.21 %ID/g for ^99m^Tc-TMHEA and ^99m^Tc-TMHHA, respectively. The tumor uptake value was higher than that of muscle, and the uptake ratio of tumor to muscle was increased with time for ^99m^Tc-TMHEA and ^99m^Tc-TMHHA, respectively. In previous work of our group [12,13], it was found that the uptake ratio of tumor to muscle increases with the increasing carbon chain length between the thymidine and N_2_S_2_ ligand (*i.e.*, from ^99m^Tc-ANMdU to ^99m^Tc-NHT). However, in the present work the ratio of ^99m^Tc-TMHHA was samller than that of ^99m^Tc-TMHEA (see Table 3), and the both ratios of ^99m^Tc-TMHHA and ^99m^Tc-TMHEA were smaller than that of ^99m^Tc-NHT (4.41 ± 0.32, at 2 h post injection) [13]. This indicates that limitless extension of the carbon chain is not always beneficial to improve the uptake ratio of tumor to muscle. The uptake ratio of tumor to bone of ^99m^Tc-TMHHA was decreased with time. The uptake ratio of tumor to blood was increased with time for ^99m^Tc-TMHEA and ^99m^Tc-TMHHA, respectively.

In summary, ^99m^Tc-TMHEA and ^99m^Tc-TMHHA had similar biological behavior, however, the uptake ratios of tumor to muscle, tumor to bone and tumor to blood of ^99m^Tc-TMHEA and ^99m^Tc-TMHHA were smaller than those of ^99m^Tc-NHT, which suggests that limitless extension of the carbon chain is not always beneficial to improve the uptake ratios of tumor to muscle, tumor to bone and tumor to blood.

**Table 3:** Biodistribution of ^99m^Tc-TMHEA and ^99m^Tc-TMHHA in mice (mean ± SD, n = 5,%ID/g).

Tissue	Time (min)						
	5	10	15	30	60	120	180
^99m^Tc-TMHEA							
Brain	0.27 ± 0.01	0.24 ± 0.07	0.20 ± 0.04	0.13 ± 0.07	0.04 ± 0.01	0.02 ± 0.01	0.02 ± 0.00
Heart	3.61 ± 0.14	2.32 ± 0.63	2.04 ± 0.11	1.01 ± 0.46	0.42±0.07	0.23 ± 0.04	0.18 ± 0.03
Liver	5.71 ± 0.81	4.83±0.87	5.22 ± 0.86	4.25 ± 0.36	4.17 ± 0.19	2.72 ± 0.44	3.27 ± 0.18
Spleen	2.42 ± 0.03	1.69 ± 0.59	1.62 ± 0.09	1.06 ± 0.34	0.79 ± 0.15	0.81 ± 0.30	0.79 ± 0.21
Lung	5.59 ± 0.73	4.15 ± 0.48	4.53 ± 0.95	2.30 ± 0.66	1.45 ± 0.20	1.50 ± 0.46	0.95 ± 0.22
Kidney	23.99 ± 0.79	15.50 ± 0.42	14.20 ± 0.50	12.45 ± 0.81	10.22 ± 0.74	8.87 ± 0.44	9.32 ± 0.24
Tumor	2.51 ± 0.28	2.00 ± 0.53	1.94 ± 0.31	1.34 ± 0.24	0.62 ± 0.12	0.51 ± 0.05	0.48 ± 0.07
Stomach	1.57 ± 0.58	1.17 ± 0.37	1.35 ± 0.24	0.73 ± 0.12	0.47 ± 0.12	1.82 ± 0.79	1.17 ± 0.29
S. intestines	3.82 ± 0.99	3.16 ± 0.57	2.90 ± 0.10	2.45 ± 0.36	2.36 ± 0.41	2.31 ± 0.03	1.98 ± 0.28
Bladder	14.18 ± 0.87	12.29 ± 0.52	10.51 ± 0.55	7.09 ± 0.04	4.24 ± 0.38	1.56 ± 0.62	1.17 ± 0.87
Muscle	1.93 ± 0.16	1.31 ± 0.41	1.55 ± 0.56	0.66 ± 0.15	0.38 ± 0.06	0.22 ± 0.08	0.14 ± 0.05
Bone	2.02 ± 0.14	1.30 ± 0.48	1.57 ± 0.52	0.87 ± 0.16	0.63 ± 0.23	0.51 ± 0.17	0.33 ± 0.15
Blood	3.86 ± 0.60	2.77 ± 0.91	2.48 ± 0.73	1.02 ± 0.25	0.48 ± 0.09	0.29 ± 0.05	0.29 ± 0.06
Tumor/Muscle	1.30 ± 0.04	1.54 ± 0.08	1.56 ± 0.02	1.98 ± 0.21	1.85 ± 0.02	2.84 ± 0.76	2.98 ± 0.37
Tumor/Bone	1.24 ± 0.06	1.59 ± 0.22	1.51 ± 0.03	1.46 ± 0.17	1.14 ± 0.12	1.27 ± 0.20	1.62 ± 0.18
Tumor/Blood	0.65 ± 0.03	0.78 ± 0.12	0.93 ± 0.01	1.21 ± 0.07	1.27 ± 0.22	1.87 ± 0.02	1.80 ± 0.41
**^99m^Tc-TMHHA**							
Brain	0.27 ± 0.02	0.25 ± 0.03	0.20 ± 0.04	0.13 ± 0.04	0.11 ± 0.04	0.03 ± 0.00	0.03 ± 0.00
Heart	3.01 ± 0.29	2.44 ± 0.10	1.79 ± 0.42	1.10 ± 0.30	0.96 ± 0.09	0.25 ± 0.05	0.23 ± 0.03
Liver	8.79 ± 0.60	8.21 ± 0.30	8.08 ± 0.66	5.43 ± 0.01	5.39 ± 0.28	4.43 ± 0.83	4.13 ± 0.34
Spleen	2.41 ± 0.30	2.13 ± 0.47	1.84 ± 0.43	1.42 ± 0.17	1.19 ± 0.20	0.83 ± 0.17	0.90 ± 0.13
Lung	4.55 ± 0.77	4.42 ± 0.04	2.96 ± 0.43	2.04 ± 0.48	1.76 ± 0.55	0.86 ± 0.15	0.75 ± 0.34
Kidney	15.38 ± 0.52	14.14 ± 0.95	9.46 ± 0.19	7.65 ± 0.39	5.83 ± 0.45	3.00 ± 0.74	3.27 ± 0.36
Tumor	2.38 ± 0.41	2.23 ± 0.60	1.43 ± 0.20	1.26 ± 0.12	0.88 ± 0.22	0.42 ± 0.12	0.41 ± 0.10
Stomach	1.55 ± 0.32	1.52 ± 0.43	0.81 ± 0.20	0.80 ± 0.16	0.62 ± 0.18	0.50 ± 0.20	0.46 ± 0.08
S. intestines	5.97 ± 0.79	5.73 ± 0.01	4.32 ± 0.42	4.00 ± 0.19	2.06 ± 0.21	0.89 ± 0.35	0.85 ± 0.17
Bladder	16.05 ± 0.95	14.47 ± 0.32	8.66 ± 0.98	4.95 ± 0.27	2.82 ± 0.56	1.43 ± 0.35	1.05 ± 0.06
Muscle	1.75 ± 0.21	1.67 ± 0.43	1.00 ± 0.23	0.65 ± 0.17	0.49 ± 0.20	0.17 ± 0.04	0.14 ± 0.02
Bone	2.27 ± 0.67	2.20 ± 0.53	1.71 ± 0.65	0.99 ± 0.21	0.79 ± 0.14	0.47 ± 0.14	0.52 ± 0.05
Blood	3.81 ± 0.47	3.37 ± 0.14	2.12 ± 0.52	1.14 ± 0.31	0.83 ± 0.22	0.31 ± 0.06	0.27 ± 0.03
Tumor/Muscle	1.36 ± 0.15	1.35 ± 0.18	1.34 ± 0.21	1.60 ± 0.08	1.62 ± 0.12	2.42 ± 0.33	2.46 ± 0.24
Tumor/Bone	1.09 ± 0.20	1.07 ± 0.12	1.06 ± 0.11	1.03 ± 0.06	0.98 ± 0.05	0.90 ± 0.16	0.81 ± 0.28
Tumor/Blood	0.72 ± 0.10	0.74 ± 0.09	0.67 ± 0.12	0.96 ± 0.04	1.07 ± 0.05	1.28 ± 0.22	1.34 ± 0.08

### 2.5. Abnormal Toxicity Test

The abnormal toxicity test was evaluated by the death and 48-h survival of the mice, which were injected with 0.2 mL ^99m^Tc-TMHEA and ^99m^Tc-TMHHA (3.7 MBq), respectively. Saline-injected (of the same volume) mouse group was used as the control group. As expected, the mice showed no signs of toxicity through the overall study period.

## 3. Experimental

### 3.1. General

All analytical chemical reagents employed were purchased from commercial sources and used without further purification. Na^99m^TcO_4_ was supplied by Jiangsu Institute of Nuclear Medicine. Electron spray ion (ESI) mass spectra were measured using a Waters Platform ZMD4000 LC/MS. NMR spectra were obtained on a Bruker DRX-500 spectrometer, and the chemical shift value was given relative to the internal tetramethylsilane (TMS). A Packard multi-prias γ Counter was used. The animal experiments in this study were approved by the Animal Care and Ethnics Committee of Jiangsu Institute of Nuclear Medicine.

### 3.2. Synthesis of ^99m^Tc-TMHEA and ^99m^Tc-TMHHA

**Scheme 1:**
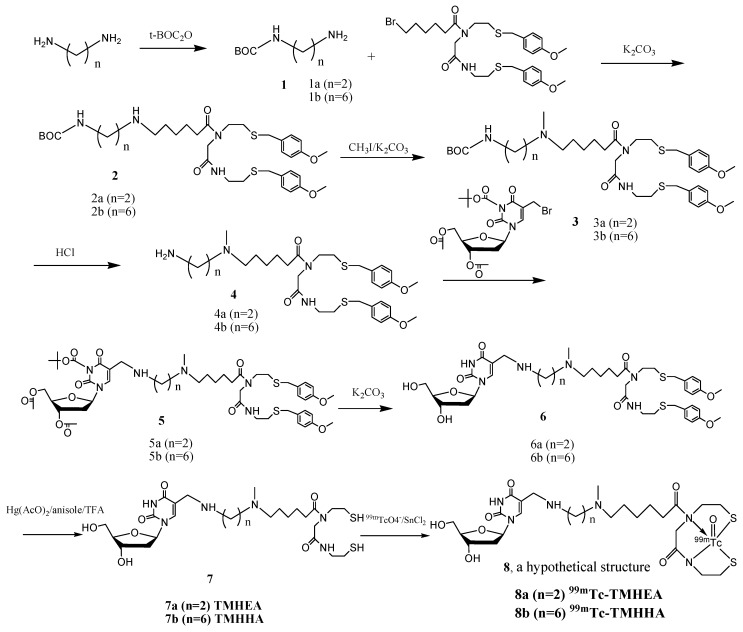
Syntheses of ^99m^Tc-TMHEA and ^99m^Tc-TMHHA.

^99m^Tc-TMHEA and ^99m^Tc-TMHHA were synthesized according to the synthetic route summarized in Scheme 1.

#### 3.2.1. General Procedure for the Preparation of Compounds **1a** and **1b**

The solution of corresponding diamine (0.5 mol) in methanol (200 mL) was cooled down to 0 °C and *t*-butoxycarbonyl anhydride (*t*-BOC_2_O, 10.8 g, 50 mmol) in methanol (10 mL) was added dropwise. The reaction mixture was stirred for 20 h at room temperature. The reaction mixture was concentrated and diluted with water. The mixture was then extracted with CH_2_Cl_2_ (60 mL) for three times. The organic layer was dried with anhydrous Na_2_SO_4_ and the solvent was evaporated to give compound **1**.

*N-BOC-ethanediamine* (**1a**): Yield: 67%. ESI-MS, *m/z* (%): 161 (100) = [M+H^+^]; ^1^H-NMR (CDCl_3_): δ 3.23–2.26 (m, 2H), 2.90–2.92 (m, 2H), 2.10–2.15 (m, 1H), 1.39–1.41 (m, 9H); ^13^C-NMR (CDCl_3_): δ (ppm) 156.0, 79.5, 43.3, 40.5, 28.5.

*N-BOC-ethanediamine* (**1b**): Yield: 85%. ESI-MS, *m/z* (%): 216 (100) = [M+H^+^]; ^1^H-NMR (CDCl_3_): δ 2.90–3.02 (m, 2H), 2.60–2.70 (m, 2H), 1.98–2.02 (m, 2H), 1.53–1.57 (m, 4H), 1.39–1.41 (s, 9H), 1.28–1.31 (m, 4H); ^13^C-NMR (CDCl_3_): δ (ppm) 156.0, 79.5, 42.1, 41.9, 32.8, 30.0, 28.5, 26.5.

#### 3.2.2. General Procedure for the Preparation of Compounds **2a** and **2b**

Compound **1** (2.0 mmol), potassium carbonate (2 g, 14.49 mmol) and *N*-{[2-(2-(S-(4-methoxybenzyl)sulfanyl)ethylamino)acetyl]-S-(4-methoxybenzyl)-2-aminoethylsulfanyl}-6-bromo-1-hexanamide (1 g, 1.6 mmol) were dissolved in an acetone/DMF (1/1, v/v) mixed solvent (100 mL), and the mixture was heated at 80 °C and stirred for 20 h under nitrogen atmosphere. Then the solvent was evaporated under reduced pressure and the desired product (compound **2**) was purified by silica gel column chromatography using ethyl acetate/methanol = 4/1 (v/v).

*N-BOC-N′-{N′′-[2-(2-(S-(4-Methoxybenzyl)sulfanyl)ethylamino)acetyl]-S-(4-methoxybenzyl)-2-aminoethylsulfanyl-1-hexanamide}-ethanediamine* (**2a**): Yield: 93%. ESI-MS, *m/z* (%): 691 (70) = [M+H]^+^, 591 (100) = [M+H−BOC]^+^, ^1^H-NMR (CDCl_3_): δ 7.18–7.24 (m, 4H), 6.81–6.88 (m, 4H), 5.02 (s, 1H), 3.85–3.89 (d, 2H, *J* = 12 Hz), 3.78 (s, 6H), 3.67–3.72 (d, 2H, *J* = 16 Hz), 3.64 (s, 2H), 3.32–3.42 (m, 4H), 3.13–3.21 (m, 2H), 2.70 (t, 2H), 2.55–2.59 (m, 4H), 2.48–2.54 (m, 2H), 2.20–2.29 (m, 2H), 1.42–1.50 (m, 16H), 1.28–1.34 (m, 2H); ^13^C-NMR (CDCl_3_): δ (ppm) 173.8, 173.6, 169.2, 168.1, 158.9, 158.7, 155.9, 130.1, 129.7, 114.1, 113.8, 79.2, 77.3, 76.9, 76.0, 55.0, 52.5, 50.9, 49.5, 49.2, 46.5, 41.5, 40.5, 37.8, 35.8, 35.0, 33.1, 32.7, 30.8, 29.8, 29.3, 28.8, 28.0, 26.8, 25.0.

*N-BOC-N′-{N′′-[2-(2-(S-(4-Methoxybenzyl)sulfanyl)ethylamino)acetyl]-S-(4-methoxybenzyl)-2-aminoethylsulfanyl-1-hexanamide}-hexamethylene diamine* (**2b**): Yield: 84%. ESI-MS, *m/z* (%): 747 (100) = [M+H^+^], ^1^H-NMR (CDCl_3_): δ 7.17–7.22 (m, 4H), 6.80–6.88 (m, 4H), 6.65–6.70 (s, 1H), 4.50 (s, 1H), 3.82–3.90 (d, 2H, *J* = 12 Hz), 3.78 (s, 6H), 3.72(s, 2H), 3.62–3.69 (d, 2H, *J* = 8 Hz), 3.50–3.55 (m, 1H), 3.32–3.46 (m, 4H), 3.08–3.12 (d, 2H, *J* = 8 Hz), 2.55–2.62 (m, 6H), 2.48–2.52 (m, 2H), 2.20–2.30 (m, 2H), 1.55–1.68 (m, 4H), 1.39–1.50 (m, 13H), 1.26–1.36 (s, 6H); ^13^C-NMR (CDCl_3_): δ (ppm) 173.8, 169.2, 168.8, 164.2, 158.9, 158.7, 156.1, 129.2, 128.8, 113.8, 113.6, 77.1, 76.8, 55.2, 52.8, 51.5, 50.5, 50.1, 49.5, 46.8, 40.5, 37.8, 37.6, 36.1, 35.1, 34.9, 33.0, 32.7, 30.8, 30.0, 29.8, 29.8, 28.8, 27.1, 26.9, 26.8, 25.0, 24.8.

#### 3.2.3. General Procedure for the Preparation of Compounds **3a** and **3b**

A mixture of compound **2** (1.5 mmol) and potassium carbonate in acetone (30 mL), and iodomethane (95 μL, 1.5 mmol) in acetone (20 mL) was added. The reaction mixture was stirred for 2 h, then the solvent was evaporated under reduced pressure. The desired product (compound **3**) was purified by silica gel column chromatography using ethyl acetate/methanol/triethylamine = 19/2/1 (v/v).

*N-BOC-N′-Methyl-N′-{N′′-[2-(2-(S-(4-methoxybenzyl)sulfanyl)ethylamino)acetyl]-S-(4methoxybenzyl)-2-aminoethylsulfanyl-1-hexanamide}-ethanediamine* (**3a**): Yield: 86%. ESI-MS, *m/z* (%): 705 (100) = [M+H^+^], ^1^H-NMR (CDCl_3_): δ 7.19–7.25 (m, 4H), 6.82–6.88 (m, 4H), 3.87–3.92 (d, 2H, *J* = 16 Hz), 3.78 (s, 6H), 3.67–3.73 (d, 2H, *J* = 16 Hz), 3.65 (s, 2H), 3.48–3.52 (m, 1H), 3.32–3.45 (m, 4H), 3.12–3.21 (m, 2H), 2.57–2.61 (m, 2H), 2.48–2.54 (m, 2H), 2.40–2.45 (m, 2H), 2.28–2.34 (m, 2H), 2.22–2.28 (m, 2H), 2.18 (s, 3H), 1.51–1.52 (m, 5H), 1.49 (s, 11H), 1.47–1.48 (m, 2H); ^13^C-NMR (CDCl_3_): δ (ppm) 173.7, 173.5, 169.2, 168.1, 158.9, 158.7, 156.0, 130.2, 129.4, 114.1, 113.8, 78.9, 77.4, 76.9, 76.1, 57.3, 56.5, 55.0, 52.1, 50.7, 49.1, 47.1, 41.8, 37.8, 36.1, 35.3, 35.1, 33.1, 32.7, 30.9, 29.2, 28.8, 28.0, 26.9, 25.0, 24.8.

*N-BOC-N′-Methyl-N′-{N′′-[2-(2-(S-(4-methoxybenzyl)sulfanyl)ethylamino)acetyl]-S-(4-methoxybenzyl)-2-aminoethylsulfanyl-1-hexanamide}-hexamethylenediamine* (**3b**): Yield: 82%. ESI-MS, *m/z* (%): 761 (100) = [M+H^+^], ^1^H-NMR (CDCl_3_): δ 7.19–7.24 (m, 4H), 6.82–6.88 (m, 4H), 3.87–3.91 (d, 2H, *J* = 12 Hz), 3.78 (s, 6H), 37.0–37.3 (s, 2H), 3.64–3.69 (d, 2H, *J* = 8 Hz), 3.51–3.56 (m, 1H), 3.32–3.45 (m, 4H), 3.03–3.13 (d, 2H, *J* = 8 Hz), 2.54–2.62 (m, 2H), 2.48–2.53 (m, 2H), 2.22–2.34 (m, 6H), 2.18 (s, 3H), 1.58–1.68 (m, 4H), 1.42–1.50 (s, 13H), 1.34–1.38 (m, 6H); ^13^C-NMR (CDCl_3_): δ (ppm) 174.0, 169.2, 168.1, 159.0, 158.8, 156.0, 130.0, 129.4, 114.0, 113.8, 78.9, 77.4, 76.9, 76.1, 57.6, 57.4, 55.5, 52.2, 51.1, 49.6, 46.9, 41.8,40.5, 37.8, 37.6, 36.0, 35.3, 35.1, 33.1, 32.7, 31.0, 29.9, 29.5, 29.2, 28.7, 28.1, 27.0, 26.3, 25.1, 25.0.

#### 3.2.4. General Procedure for the Preparation of Compounds **4a** and **4b**

The solution of compound **3** (1.3 mmol) in methanol (50 mL), and concentrated hydrochloric acid (5 mL) was added. The reaction mixture was stirred for 4 h at 45 °C, then the solvent was evaporated under reduced pressure. The residue was dissolved in water (30 mL) and the mixture was adjusted to pH 10 by aqueous sodium hydroxide solution (2 mol/L). The mixture was then extracted with ethyl acetate (30 mL) for three times. The organic layer was dried with anhydrous Na_2_SO_4_ and the solvent was evaporated under reduced pressure. The desired product (compound **4**) was purified by silica gel column chromatography using ethyl acetate/methanol/triethylamine = 8/8/1 (v/v).

*N-Methyl-N-{N′-[2-(2-(S-(4-methoxybenzyl)sulfanyl)ethylamino)acetyl]-S-(4-methoxybenzyl)-2-aminoethylsulfany-1-hexanamide}-ethanediamine* (**4a**): Yield: 84%. ESI-MS, *m/z* (%): 605 (100) = [M+H^+^], ^1^H-NMR (CDCl_3_): δ 7.17–7.23 (m, 4H), 6.78–6.85 (m, 4H), 5.28 (s, 1H), 3.87–3.92 (d, 2H, *J* = 16 Hz), 3.78 (s, 6H), 3.62–3.70 (m, 4H), 3.51–3.57 (m, 1H), 3.31–3.45 (m, 4H), 2.71–2.80 (m, 3H), 2.50–2.61 (m, 4H), 2.38–2.42 (m, 2H), 2.30–2.38 (m, 2H), 2.20–2.28 (m, 2H), 2.15–2.18 (d, 3H, *J* = 4 Hz), 1.57–1.63 (m, 2H), 1.40–1.49 (m, 2H), 1.25–1.32 (m, 2H); ^13^C-NMR (CDCl_3_): δ (ppm) 173.1, 168.1, 158.9, 158.7, 129.5, 113.1, 77.1, 76.9, 76.0, 56.0, 55.0, 53.5, 50.5, 49.2, 46.7, 41.5, 41.0, 37.8, 37.0, 35.5, 34.8, 34.2, 32.0, 30.5, 29.0, 28.8, 26.7, 26.2, 23.8, 23.5.

*N-Methyl-N-{N′-[2-(2-(S-(4-methoxybenzyl)sulfanyl)ethylamino)acetyl]-S-(4-methoxybenzyl)-2-aminoethylsulfanyl-1-hexanamide}-hexamethylenediamine* (**4b**): Yield: 89%. ESI-MS, *m/z* (%): 661 (100) = [M+H^+^], ^1^H-NMR (CDCl_3_): δ 7.19–7.25 (m, 4H), 6.81–6.88 (m, 4H), 3.87–3.92 (d, 2H, *J* = 16 Hz), 3.78 (s, 6H), 3.64–3.72 (m, 4H), 3.51–3.57 (m, 1H), 3.29–3.45 (m, 4H), 2.88–2.94 (s, 3H), 2.68–2.72 (m, 2H), 2.48–2.60 (m, 4H), 2.33–2.38 (m, 4H), 2.24–2.30 (m, 2H), 2.22 (s, 3H), 1.94 (s, 1H), 1.58–1.66 (m, 2H), 1.42–1.52 (m, 4H), 1.28–1.38 (m, 6H); ^13^C-NMR (CDCl_3_): δ (ppm) 178.1, 173.1, 169.1, 168.2, 158.9, 158.7, 129.6, 113.8, 77.5, 76.9, 76.0, 57.2, 57.0, 55.2, 53.3, 52.2, 50.8, 49.8, 46.7, 41.8, 41.2, 37.8, 35.8, 35.3, 35.1, 32.8, 32.5, 31.5, 30.5, 29.5, 28.8, 27.0, 26.2, 25.0, 24.0. 

#### 3.2.5. General Procedure for the preparation of compounds **5a** and **5b**

The solution of compound **4** (0.96 mmol) and triethylamine (2 mL) in CH_2_Cl_2_ (80 mL) was cooled down to 0 °C and 3′,5′-diacetyl-5-bromo-N-BOC-thymidine (0.44 g, 0.86 mmol) in CH_2_Cl_2_ (20 mL) was added. The reaction mixture was stirred for 4 h at room temperature. The mixture was washed with aqueous NaHCO_3_ (75 mL × 2) and H_2_O (50 mL), respectively. The organic layer was dried with anhydrous Na_2_SO_4_ and the solvent was evaporated. The residue was purified by column chromatography using ethyl acetate/ methanol/triethylamine (19/1/1, v/v) to give compound **5**.

*N-(3′,5′-Diacetyl-N′-BOC-thymidinyl)-N′′-methyl-N′′-{N′′′-[2-(2-(S-(4-methoxybenzyl)sulfanyl)ethyl amino)acetyl]-S-(4-methoxybenzyl)-2-aminoethylsulfanyl-1-hexanamide}-ethanediamine* (**5a**): Yield: 51%. ESI-MS, *m/z* (%): 1029 (100) = [M+H^+^], ^1^H-NMR (CDCl_3_): δ 7.55 (s, 1H), 7.17–7.23 (m, 4H), 6.81–6.87 (m, 4H), 6.28 (m, 1H), 5.19 (m, 1H), 4.28–4.40 (m, 2H), 4.21 (s, 1H), 3.85–3.91 (d, 2H, *J* = 16 Hz), 3.78 (s, 6H), 3.61–3.71 (m, 4H), 3.55 (s, 2H), 3.30–3.50 (m, 4H), 2.65–2.70 (s, 2H), 2.55–2.60 (m, 2H), 2.45–2.51 (m, 2H), 2.30–2.35 (m, 2H), 2.20–2.30 (m, 4H), 2.18 (s, 3H), 2.02–2.10 (d, 3H, *J* = 16 Hz), 1.72–1.88 (m, 6H), 1.60 (s, 9H), 1.38–1.47 (m, 4H), 1.25–1.30 (m, 2H); ^13^C-NMR (CDCl_3_): δ (ppm) 171.4, 170.8, 170.3, 170.2, 170.1, 162.3, 159.1, 154.3, 149.7, 136.5, 130.0, 129.8, 129.8, 129.7, 129.3, 114.3, 114.2, 114.1, 109.9, 84.9, 83.3, 78.8, 74.4, 62.2, 55.9, 52.9, 49.7, 48.8, 46.6, 43.4, 39.9, 39.1, 38.7, 36.8, 33.6, 31.7, 28.9, 28.7, 28.3, 27.4, 26.4, 25.7, 21.0, 20.7.

*N-(3′,5′-Diacetyl-N′-BOC-thymidinyl)-N′′-methyl-N′′-{N′′′-[2-(2-(S-(4-methoxybenzyl)sulfanyl)ethyl amino)acetyl]-S-(4-methoxybenzyl)-2-aminoethylsulfanyl-1-hexanamide}-hexamethylenediamine* (**5b**): Yield: 48%. ESI-MS, *m/z* (%): 1085 (100) = [M+H^+^], ^1^H-NMR (CDCl_3_): δ 7.54 (s, 1H), 7.18–7.24 (m, 4H), 6.80–6.87 (m, 4H), 6.29 (m, 1H), 5.20 (m, 1H), 4.27–4.38 (m, 2H), 4.20 (s, 1H), 3.83–3.90 (d, 2H, *J* = 16 Hz), 3.78 (s, 6H), 3.60–3.71 (m, 4H), 3.55 (s, 2H), 3.30–3.50 (m, 4H), 2.65–2.70 (s, 2H), 2.55–2.60 (m, 2H), 2.45–2.51 (m, 2H), 2.30–2.35 (m, 2H), 2.20–2.30 (m, 4H), 2.18 (s, 3H), 2.02–2.10 (d, 3H, *J* = 16 Hz), 1.72–1.88 (m, 6H), 1.60 (s, 9H), 1.39–1.48 (m, 8H), 1.26–1.30 (m, 6H); ^13^C-NMR (CDCl_3_): δ (ppm) 171.4, 170.8, 170.3, 170.2, 170.1, 162.3, 159.1, 154.3, 149.7, 136.5, 130.0, 129.8, 129.8, 129.7, 129.3, 114.3, 114.2, 114.1, 109.9, 84.9, 83.3, 78.8, 74.4, 62.2, 56.9, 55.9, 52.9, 49.7, 49.6, 48.8, 46.6, 43.4, 39.9, 39.1, 38.7, 36.8, 33.6, 31.7, 30.7, 28.9, 28.7, 28.3, 28.1, 27.4, 27.1, 26.8, 26.5, 25.5, 20.8, 20.5.

#### 3.2.6. General Procedure for the Preparation of Compounds **6a** and **6b**

The mixture of compound **5** (0.45 mmol) and potassium carbonate (1 g, 7.25 mmol) in methanol (50 mL) was heated to reflux for 4 h. Then the solvent was evaporated under reduced pressure and the residue was dissolved in chloroform (50 mL). The organic layer was washed with H_2_O (50 mL) and dried with anhydrous Na_2_SO_4_. The compound **6** was purified by silica gel column chromatography using chloroform /methanol/triethylamine (5/2/0.1, v/v).

*N-Thymidinyl-N′-methyl-N′-{N′′-[2-(2-(S-(4-methoxybenzyl)sulfanyl)ethylamino)acetyl]-S-(4-methoxy-benzyl)-2-aminoethylsulfanyl-1-hexanamide}-ethanediamine* (**6a**): Yield: 68%. ESI-MS, *m/z*(%): 845 (100) = [M+H^+^], ^1^H-NMR (CDCl_3_): δ 8.05 (d, 1H, *J* = 4 Hz), 7.18–7.25 (m, 4H), 6.80–6.87 (m, 4H), 6.19–6.24 (m, 1H), 4.45 (s, 1H), 4.00–4.20 (s, 5H), 3.90–4.00 (d, 2H, *J* = 12 Hz), 3.82–3.88 (d, 2H, *J* = 8 Hz), 3.78 (s, 6H), 3.60–3.70 (m, 4H), 3.28–3.50 (m, 6H), 2.65 2013;2.72 (d, 2H, *J* = 8 Hz), 2.40–2.60 (m, 5H), 2.20–2.35 (m, 5H), 2.15 (s, 6H), 1.51–1.60 (m, 2H), 1.40–1.49 (m, 2H), 1.21–1.32 (m, 2H); ^13^C-NMR (CDCl_3_): δ (ppm) 207.1, 174.6, 174.4, 169.0, 168.5, 164.0, 158.3, 150.8, 138.2, 130.3, 114.0, 111.8, 87.1, 85.5, 77.0, 76.8, 76.5, 69.8, 61.2, 57.9, 56.8, 55.5, 52.9, 51.0, 49.5, 47.0, 46.0, 45.1, 42.0, 40.7, 37.9, 35.9, 35.0, 33.1, 32.8, 31.0, 29.3, 28.5, 27.0, 26.5, 24.5.

*N-Thymidinyl-N′-methyl-N′-{N′′-[2-(2-(S-(4-methoxybenzyl)sulfanyl)ethylamino)acetyl]-S-(4-methoxy-benzyl)-2-aminoethylsulfanyl-1-hexanamide}-hexamethylenediamine* (**6b**): Yield: 67%. ESI-MS, *m/z* (%): 901 (100) = [M+H^+^], ^1^H-NMR (CDCl_3_): δ 8.42 (s, 1H), 7.19–7.24 (d, 4H, *J* = 8 Hz), 6.79–6.86 (m, 4H), 5.51 (s, 2H), 4.45 (s, 1H), 3.90–4.02 (m, 4H), 3.78 (s, 6H), 3.65–3.72 (m, 4H), 3.50–3.61 (m, 7H), 3.40–3.45 (m, 2H), 3.32–3.37 (m, 1H), 2.95–3.07 (m, 11H), 2.75 (s, 2H), 2.51–2.60 (m, 3H), 2.25–2.38 (m, 2H), 1.58–1.62 (m, 2H), 1.32–1.48 (m, 12H); ^13^C-NMR (CDCl_3_): δ (ppm) 205.1, 173.6, 173.4, 168.8, 168.3, 163.8, 158.1 150.6, 138.1, 130.1, 113.9, 111.6, 87.1, 85.3, 77.2, 76.9, 76.7, 57.0, 56.8, 55.2, 53.3, 52.2, 50.8, 49.8, 46.5, 41.8, 41.2, 37.8, 35.8, 35.3, 35.0, 32.8, 32.5, 31.5, 30.5, 29.5, 28.8, 26.8, 26.2, 24.8, 23.9.

#### 3.2.7. General Procedure for the Preparation of Compounds **7a** and **7b**

Compound **6** (0.17 mmol) was dissolved in trifluoroacetic acid (5 mL) and cooled in ice bath to 0 °C. Anisole (0.26 mL) and Hg(AcO)_2_ (0.17 g, 0.53 mmol) were added. The reaction mixture was stirred for 30 min at room temperature and then concentrated in vacuum to obtain viscous brown oil that was dried in vacuum for 30 min. Dry diethyl ether (15 mL) was added to the oil and the resultant suspension stirred for about 10min. The ether was decanted and the precipitate washed again with another 15 mL of ether. The colorless solid was collected by suction filtration, dried in vacuum and dissolved again in absolute ethanol (10 mL). H_2_S gas was passed through the solution for 20 min. The reaction mixture was filtered and the filtrate concentrated under vacuum to afford compound **7** as a colorless oil.

*N-Thymidinyl-N′-methyl-N′-{N′′-[2-sulfanylethylamino)acetyl]-2-aminoethylsulfanyl-1-hexanamide}- ethanediamine* (**7a**,TMHEA): Yield: 70%. ESI-MS, *m/z* (%): 605 (100) = [M+H^+^], ^1^H-NMR (D_2_O): δ 8.18 (s, 1H), 6.24 (m, 1H), 4.49 (m, 1H), 4.07 (m, 1H), 3.98 (d, 2H, *J* = 12 Hz), 3.82–3.88 (m, 1H), 3.72–3.79 (m, 2H), 3.61–3.67 (m, 2H), 3.45–3.52 (m, 2H), 3.29–3.36 (m, 2H), 3.15–3.23 (m, 6H), 3.03–3.10 (m, 7H), 2.82 (m, 3H), 2.70–2.72 (m, 2H), 2.32–2.48 (m, 3H), 1.60–1.82 (m, 4H), 1.38–1.42 (m, 2H), 1.13–1.15 (t, 1H), 1.06–1.11 (t, 1H); ^13^C-NMR (D_2_O): δ (ppm) 164.8, 151.8, 145.0, 143.5, 104.8, 104.0, 87.0, 85.9, 70.4, 62.8, 61.1, 57.7, 55.9, 47.0, 43.9, 39.9, 39.1, 33.8, 25.7, 25.5, 24.4, 23.5, 23.1, 16.8, 10.0.

*N-Thymidinyl-N′-methyl-N′-{N′′-[2-sulfanylethylamino)acetyl]-2-aminoethylsulfanyl-1-hexanamide}- hexamethylenediamine* (**7b**,TMHHA): Yield: 75%. ESI-MS, *m/z* (%): 661 = [M+H^+^], ^1^H-NMR (D_2_O): δ 8.20 (s, 1H), 6.22–6.35 (m, 1H), 4.49 (m, 1H), 4.03–4.18 (m, 4H), 3.78–3.92 (m, 2H), 3.45–3.67 (m, 2H), 3.10–3.38 (m, 6H), 2.93–3.02 (s, 6H), 2.70–2.90 (m, 5H), 2.32–2.48 (m, 3H), 2.22 (s, 3H), 1.95–2.05 (m, 2H), 1.61–1.85 (m, 10H), 1.38–1.45 (m, 4H), 1.23–1.25 (t, 1H), 1.16–1.21 (t, 1H); ^13^C-NMR (D_2_O): δ (ppm) 165.9, 164.5, 151.0, 144.1, 104.6, 87.0, 86.5, 70.3, 62.5, 61.0, 56.8, 51.0, 48.0, 47.0, 44.7, 43.0, 41.1, 40.1, 39.2, 37.0, 33.9, 33.2, 30.0, 29.2, 25.0, 24.1, 23.5, 23.0, 22.0.

### 3.3. Radiochemical Syntheses of ^99m^Tc-TMHEA *(**8a**)* and ^99m^Tc-TMHHA *(**8b**)*

A solution of compound **7** (50 μL, 2 mg of compound 7 dissolved in 2 mL ethanol) was added to a mixture of sodium glucoheptonate (0.8 mL, 10 mg/mL), freshly prepared solution of stannous chloride dehydrate (20 μL, 1.0 mg SnCl_2_.2H_2_O dissolved in 1 mL 0.1 mol/L hydrochloric acid solution), and pertechnetate eluate (50 μL, 37 MBq). The reaction mixture was vortexed adequately and reacted at 100 °C for 30 min.

### 3.4. Quality Control of ^99m^Tc-TMHEA and ^99m^Tc-TMHHA

The radiochemical purity (RCP) and radiolabeling yield (RLY) of ^99m^Tc-TMHEA and ^99m^Tc-TMHHA was determined by TLC and HPLC.

#### 3.4.1. TLC

About 3 μL ^99m^Tc-TMHEA and ^99m^Tc-TMHHA solutions were spotted with a glass capillary at 1.2 cm from the bottom of polyamide layer strips. The polyamide layer strips were eluted by ascending chromatography with toluene/acetonitrle/methanol (3/1/1, v/v). The ^99m^Tc-colloidal impurities remain at the bottom on polyamide layer strip, while **8a** and **8b** both migrate with the solvent front. The strips were cut into pieces of 1 cm and the activity of these pieces was counted to determine the RCP value on a well-type γ counter.

#### 3.4.2. HPLC

The RCP of ^99m^Tc-TMHEA and ^99m^Tc-TMHHA were determined by HPLC using a Waters 600-type instrument. The sample was carefully passed through a Millipore filter and injected into the HPLC column (SunFire^TM^ C18, PN: 186002559, 4.6 mm × 150 mm × 5 μm, Waters, Milford, MA, USA). Radioanalysis of the labeled compound was conducted using a Cd (Te) detector. The flow rate was adjusted to 1.0 mL/min and the isocratic mobile phase was 68% water and 32% methanol.

### 3.5. *In Vitro* Stability of ^99m^Tc-TMHEA and ^99m^Tc-TMHHA

The *in vitro* stabilities of ^99m^Tc-TMHEA and ^99m^Tc-TMHHA were studied in PBS (pH = 7.4) after different interval (1, 2, 3, 4, 5 and 6 h) at physiological temperature of 37 °C. The RCP values were evaluated by HPLC at different time points to determine whether they were stable *in vitro*.

### 3.6. Octanol-Water Partition Coefficients of ^99m^Tc-TMHEA and ^99m^Tc-TMHHA

The partition coefficients (log*P*) of ^99m^Tc-TMHEA and ^99m^Tc-TMHHA were determined in *n*-octanol and two kinds of phosphate buffered saline (PBS, pH 7.0 and pH 7.4, respectively). For each pH, a sample of radiolabeled compound ^99m^Tc-TMHEA and ^99m^Tc-TMHHA (20 μL, 0.74 MBq) was added to the two-phase system of 3.0 mL *n*-octanol and 3.0 mL PBS, respectively. The mixture was vortexed for 1 min × 3 and centrifuged for 5 min at 4,000 r/min to ensure complete separation of layers, and then 1.0 mL *n*-octanol and 1.0 mL PBS were taken out and counted with a γ-counter. Afterwards, 1.0 mL *n*-octanol was transferred to another tube containing 3.0 mL PBS and 2.0 mL *n*-octanol. The above procedure was repeated for six times. Log*P* values were calculated using the formula of log*P* = log[counts(*n*-octanol)/counts(PBS)].

### 3.7. Tumor Models

The mouse hepatoma HepA ascites tumor cells were maintained in ICR mice by weekly intraperi- toneal transplantation into fresh ICR mice and were collected for transplantation under sterile conditions. Tumor xenografts were established in 5- to 7-week-old ICR mice (18–20 g) by injection of approximately 2 × 10^6^ HepA cells in the right shoulder area. When the tumors were about 0.8 cm in diameter (about 7 days), the mice were used for biodistribution as described below.

### 3.8. Biodistribution in Tumor-Bearing Mice of ^99m^Tc-TMHEA and ^99m^Tc-TMHHA

Thirty-five tumor-bearing mice (18 male and 17 female) were randomly divided into seven groups and injected via the tail vein with the test agent (^99m^Tc-TMHEA and ^99m^Tc-TMHHA) in a volume of 0.2 mL and activity of approximately 3.7 MBq. Groups of mice were sacrificed by decapitation at 5, 10, 15, 30, 60, 120 and 180 min after injection. The organs of interest (heart, muscle, lung, kidney, spleen, liver and tumor *etc.*) were dissected and weighed, as well as 100 μL blood were taken from carotid artery. The activity for each sample was determined by a γ counter. Distribution of the radioactivity in different tissues and organs was calculated and expressed as percentage of injection dose per gram (%ID/g).

## 4. Conclusions

TMHEA and TMHHA, two kinds of novel thymidine derivative, have been prepared and successfully labeled with ^99m^Tc in a high labeling yield and good *in vitro* stability. ^99m^Tc-TMHEA and ^99m^Tc-TMHHA had similar biological behavior, however, the uptake ratios of tumor to muscle, tumor to bone and tumor to blood of ^99m^Tc-TMHEA and ^99m^Tc-TMHHA were smaller than those of ^99m^Tc-NHT, which means limitless extension of the carbon chain is not always beneficial to improve the uptake ratios.
